# Follow-up report on pulmonary mucosa-associated lymphoma in a patient with von Hippel–Lindau disease

**DOI:** 10.1093/jscr/rjae619

**Published:** 2024-10-05

**Authors:** Bukola A Olarewaju, Judy B Tejon, Mayowa A Osundiji

**Affiliations:** School of Science and Engineering, University of Dundee, Nethergate, Dundee DD1 4HN, United Kingdom; Department of Clinical Genomics, Mayo Clinic, East Shea Blvd, Scottsdale, AZ 85259, United States; Department of Clinical Genomics, Mayo Clinic, East Shea Blvd, Scottsdale, AZ 85259, United States

**Keywords:** von Hippel–Lindau, lymphoma, tumor

## Abstract

With the increasing opportunities for medical management of von Hippel–Lindau (VHL)-related lesions, fundamental questions are arising regarding the possibilities of the FDA-approved medication (Belzutifan) to delay the progression of other non-VHL-related tumors that are not requiring urgent surgical interventions. We present a follow-up report on a VHL patient, with a co-morbid history of pulmonary mucosa-associated lymphoid tissue lymphoma, who was initially described in the clinical scientific literature ~10 years ago. This patient’s case highlights some of the challenges for some VHL patients with comorbid non-VHL-related tumors and potential opportunities for targeted support.

## Introduction

First described in the 19th century, von Hippel-Lindau (VHL) is a rare familial disorder that presents with a variety of cancerous and noncancerous tumors in the kidneys, the central nervous system, retina, adrenal gland, and pancreas [1, 2]. New therapies targeting the molecular mechanisms for VHL disease have emerged in the past decade, including Belzutifan [3] and potentially pazopanib [4]. Approved by the FDA in 2021 for the treatment of renal cell carcinoma (RCC) in patients with VHL disease, Belzutifan, is a hypoxia-inducible factor 2-alpha inhibitor that has continued to show promising responses as a potential therapeutic option for RCC and other VHL-related tumors [5–8]. Pazopanib is a multiple tyrosine kinase inhibitor that also appears quite promising for VHL-related tumors [4].

Asides from tumors that are well known to be associated with VHL, possibilities of an increased risk of other tumors in VHL patients have continued to arouse burgeoning clinical research interests [9–11]. The potential benefits of the new VHL therapies (such as Belzutifan) for non-VHL-related tumors have also continued to be subjects of active investigations [9]. Straughan and colleagues reported the first case of extranodal (pulmonary) lymphoma of mucosa-associated lymphoid tissue (MALT) in a VHL patient [11] in 2015. Considering the new and ongoing discussions of the potential significance of Belzutifan in non-VHL-related tumors, we hereby provide a follow-up report on the patient who was originally described by Straughan and colleagues [11] to highlight some important concerns and possible opportunities for targeted support for VHL patients with tumors that are not traditionally known to be associated with VHL.

## Follow-up clinical report

We provide a follow-up report on a 65-year-old female who presented to the VHL genetics clinic as part of multidisciplinary assessments. She had bilateral pheochromocytoma around age 21 that necessitated bilateral adrenalectomy. At around age 31, she had pancreatic neuroendocrine tumors requiring partial pancreatomy. She developed multiple CNS hemangioblastomas and renal cystic lesions, as well as renal cell cancer, requiring partial nephrectomy. Accordingly, the history and findings prompted a clinical diagnosis of VHL around age 47. She subsequently underwent genetic testing around age 48, which showed a heterozygous pathogenic variant in the VHL gene that is denoted as R167Q (located within exon 3) and suggestive of VHL type 2B. Around age 51, she presented with bilateral pulmonary ground glass opacities and a lesion in the right upper lobe of the lung that was reportedly biopsied and deemed to show a clonal B-cell process that is consistent with an extranodal marginal zone lymphoma of the MALT type. The lesion was successfully resected, and she recovered well. In the interim, the patient has advised that there was a discrepancy in her age that was hitherto reported [11]. The patient explained that she was around 56 years old at the time of the previous report. In nearly a decade, most of the VHL lesions have remained stable. There have also been new VHL lesions, interval worsening of some lesions, and recurrence of the pulmonary MALT lymphoma in the interim. The majority of the CNS hemangioblastomas have remained relatively stable, although newer CNS hemagioblastomas have also arisen. She has experienced recurrence of the pulmonary MALT lymphoma and pancreatic neuroendocrine tumors. There have also been interval concerns of moderate sensorineural hearing loss. She has no known history of endolymphatic sac tumors, or retinal angioma. Apart from the history of surgical interventions for VHL-related lesions, she previously underwent surgery for right hemicolectomy (due to cecal volvulus) as well as total hysterectomy with salpingo-oophorectomy and thyroidectomy plus tonsillectomy.

Possibilities of follow-up surgical management for recurrent pulmonary MALT lymphoma and/or Belzutifan therapy for VHL were discussed, but the patient had major concerns regarding her overall personal risk profile and hoped that emerging dataset may help shed more light on known outcomes for VHL patients with potentially similar co-morbidities, including non-VHL tumors. The patient was concerned about the probable risk of anemia and hypoxia that can accompany Belzutifan therapy and her ability to cope in the setting of her overall medical and surgical history, including pulmonary MALT lymphoma. Multidisciplinary molecular tumor board discussions were held, and a conservative management approach with very frequent surveillance modalities was favored during the patient’s last visit to the clinic.

## Discussion

In the interim, there have been no new reports of potential association(s) between pulmonary MALT lymphoma and VHL disease to date. Considering that neoplastic proliferation of hematopoietic cells is known to occur with the homozygous VHL gene germline c.598C > T (Arg200Trp) variant that was originally described in individuals from the Chuvash region [12, 13] and that the clonal process appears to be potentially responsive to Belzutifan therapy [9], the potential association between pulmonary MALT lymphoma and the VHL gene remains topical. An improved understanding of the molecular mechanisms underpinning the VHL gene’s role in hematological disorders is increasingly becoming crucial. Interval reports of new medications have continued to provide additional options for the clinical management of VHL-related tumors; however, further studies are increasingly needed to help facilitate decisions for patients with complex medical histories that include concomitant VHL-related and non-VHL-related tumors. Phase 1 studies on Belzutifan therapy in patients with advanced solid tumors (including non-VHL-related tumors) showed that it was well tolerated [14]. As the new medications for VHL continue to expand the potential therapeutic options for VHL patients, increased reporting of therapeutic responses and adverse effects in VHL patients will help guide medical and/or surgical management decisions in VHL patients with more complex medical histories.
